# Bacteriocin-Producing *Escherichia coli* Q5 and C41 with Potential Probiotic Properties: In Silico, In Vitro, and In Vivo Studies

**DOI:** 10.3390/ijms241612636

**Published:** 2023-08-10

**Authors:** Veronika S. Mihailovskaya, Dmitry A. Sutormin, Marina O. Karipova, Anna B. Trofimova, Victor A. Mamontov, Konstantin Severinov, Marina V. Kuznetsova

**Affiliations:** 1 Institute of Ecology and Genetics of Microorganisms, Ural Branch of the Russian Academy of Sciences, Goleva Street 13, 614081 Perm, Russia; veranikamihailovskaja@yandex.ru; 2 Skolkovo Institute of Science and Technology, 121205 Moscow, Russia; d.a.sutormin@gmail.com (D.A.S.); viktor.mamontov@skoltech.ru (V.A.M.); 3 Department of Microbiology and Virology, Perm State Medical University Named after Academician E. A. Wagner, 614000 Perm, Russia; mari.karipova@yandex.ru; 4 Institute of Gene Biology Russian Academy of Sciences, 119334 Moscow, Russia; anechka.tro@mail.ru; 5 Waksman Institute for Microbiology, Rutgers, The State University of New Jersey, Piscataway, NJ 08854, USA; severik@waksman.rutgers.edu; 6 Institute of Molecular Genetics, National Research Center “Kurchatov Institute”, 123182 Moscow, Russia

**Keywords:** *Escherichia coli*, bacteriocins, probiotic, whole genome sequencing, in silico, in vitro, in vivo, experimental infection, farm animals

## Abstract

Commensal bacteriocin-producing *Escherichia coli* are of interest for possible use as probiotics to selectively control the spread of pathogenic bacteria. Here, we evaluated the biosafety and efficacy of two new bacteriocin-producing *E. coli* strains, Q5 (VKM B-3706D) and C41 (VKM B-3707D), isolated from healthy farm animals. The genomes of both strains were sequenced, and genes responsible for the antagonistic and colonization abilities of each strain were identified. In vitro studies have shown that both strains were medium-adhesive and demonstrated antagonistic activity against most enteropathogens tested. Oral administration of 5 × 10^8^ to 5 × 10^10^ colony-forming units of both strains to rats with drinking water did not cause any disease symptoms or side effects. Short-term (5 days) oral administration of both strains protected rats from colonization and pathogenic effects of a toxigenic beta-lactam-resistant strain of *E. coli* C55 and helped preserve intestinal homeostasis. Taken together, these in silico, in vitro, and in vivo data indicate that both strains (and especially *E. coli* Q5) can be potentially used for the prevention of colibacillosis in farm animals.

## 1. Introduction

Infectious diseases cause significant economic damage to agricultural enterprises by reducing productivity and overall animal welfare [1]. Treatment of infectious diseases in farm animals is complicated by the spread of multidrug-resistance (MDR) among most pathogenic bacteria, including *Escherichia coli* [2,3]. With the growing need for the prevention and treatment of diseases caused by such strains, probiotics have gained new attention [4]. Probiotics are living microorganisms that benefit the host by improving microbial balance, regulating mucosal and systemic immunity, and antagonizing pathogenic and opportunistic microorganisms [5,6]. Probiotics are particularly advantageous as they allow for a decrease in the use of antibiotics in livestock.

Commensal *E. coli* isolates producing antimicrobial peptides (bacteriocins) can help control the spread of pathogenic enterobacteria [7]. *E. coli* is known to produce two types of bacteriocins, classified by their molecular weight into colicins (>10 kDa) and microcins (<10 kDa), with diverse mechanisms of action: pore formation (colicins A, E1, K, N, U, S4, B, Ia, Ib, microcins V and L), nuclease activity (colicins E2, E3, E4, E5, E6, E7, E8, E9, D), inhibition of peptidoglycan biosynthesis (colicin M), inhibition of DNA gyrase (microcin B17), RNA polymerase (microcin J25), an aminoacyl tRNA synthetase (microcin C7), or ATP synthase (microcin H47) [8]. The widely used probiotic “Mutaflor” contains the *E. coli* Nissle 1917 strain producing microcins M and H47 [9,10], while “Symbioflor 2” contains *E. coli* G3/10 producing microcin C7 [4]. The probiotics “Colibakterin” and “Bifikol” used in Russia are developed on the basis of *E. coli* M-17, a producer of bacteriocins B, M, and microcin V [11].

The ability of bacteriocin-producing *E. coli* to inhibit pathogens in vitro is well described in many studies [12,13]. However, only in a few studies has a direct correlation been demonstrated between the effectiveness of bacteriocin production in vitro and protection against pathogenic bacteria in vivo [14,15]. The latter is determined, at least in part, by the colonization ability caused by the adhesive activity of a microorganism. High adhesive activity allows bacteria to stay in the intestine for longer periods of time and, consequently, increases the duration of their positive effect in the gastrointestinal tract on the host microbiota and immune system [16]. An additional requirement for a probiotic strain is the absence of virulence factors and antibiotic resistance genes (ARGs) [17].

Earlier, we characterized a collection of commensal bacteriocin-producing *E. coli* strains isolated from healthy farm animals and identified two strains that hold promise for probiotic development [18]. In this study, we present a comprehensive characterization of these strains, including whole-genome sequencing and analysis, and demonstrate their efficacy against pathogens in vitro and in vivo.

## 2. Results

### 2.1. In silico Analysis of E. coli Q5 and C41 Genomes

#### 2.1.1. General Genome Features

**Figure 1:**
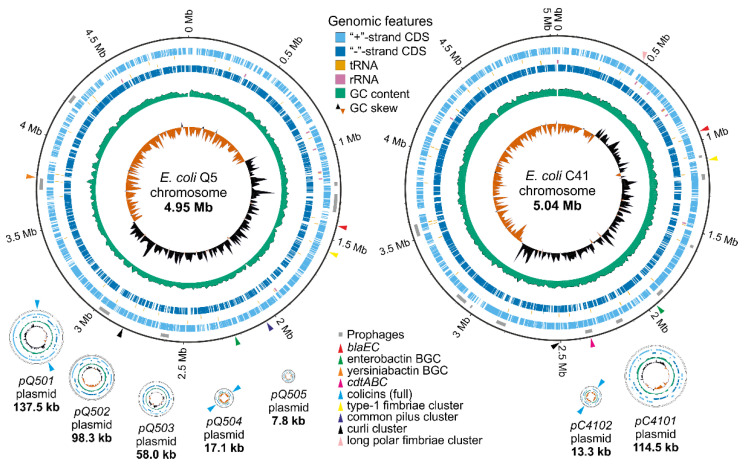
Genomes of *E. coli* Q5 and C41 strains were visualized using GenoVi [19]. Plasmids and chromosomes are not shown to scale. Grey rectangles indicate prophages; colored triangles indicate genomic features related to probiotic properties.

Complete genomes of *E. coli* Q5 and C41 were obtained by long-read sequencing using the Oxford Nanopore Technology (MinION) (Table S1). The genome of *E. coli* Q5 comprises a 4,948,409 bp chromosome with a GC content of 51.0% and five plasmids, named pQ501 (137,557 bp, GC content of 48.0%), pQ502 (98,317 bp, GC content of 48.0%), pQ503 (58,014 bp, GC content of 42.0%), pQ504 (17,107 bp, GC content of 48.0%), and pQ505 (7791 bp, GC content of 51.0%) (Figure 1). The genome of *E. coli* C41 comprises a 5,037,330 bp chromosome with a GC content of 51.3% and two plasmids, named pC4101 (114,534 bp, GC content of 48.0%) and pC4102 (13,295 bp, GC content of 49.0%). All plasmids share a high level of identity (>99%) with known sequences from the nt database (released on 4 May 2023). A total of 4945 protein-coding sequences (CDSs) were identified in the Q5 genome and 4878 CDSs—in the C41 genome. Both strains encoded 22 rRNAs, 89 tRNAs, and 1 tmRNA (Table S1). The Q5 genome shared 96.2% sequence average nucleotide identity with that of C41 (PMID: 26585518). According to PHASTEST, the Q5 genome contains seven intact prophages, whereas the C41 genome contains 10 intact and one incomplete prophages (Table S2).

Plasmids pQ501, pQ502, pQ503, and pQ504 have low (~1–2) copy-numbers, whereas pQ505 has a high copy-number (~90) (Table S3). The pQ501 and pQ503 plasmids carry the F and P-type conjugation systems, respectively. The pQ504 and pQ505 plasmids encode mobilization systems. The pQ502 plasmid was recognized as a Punavirus (Uroviricota) by BLAST (89% coverage and 99% identity with the Punavirus P1 sequence MH422554.1). With the exception of pQ505, which was not assigned to any known incompatibility group, all Q5 plasmids belong to different incompatibility groups, providing supporting evidence that they are not the result of a misassembly. Both plasmids of the C41 strain are single-copy and belong to different incompatibility groups. The pC4101 plasmid contains an F-type conjugation system, and the pC4102 plasmid has a mobilization system (Table S3).

#### 2.1.2. Antimicrobial Resistance and Virulence-Associated Genes

Mobile ARGs and virulence-associated genes (VAGs) are undesirable in probiotic strains. Prediction of ARGs with Abricate and VRprofile2 in the *E. coli* Q5 and C41 genomes revealed, respectively, chromosomal *blaEC-5* and *blaEC-18* genes encoding beta-lactamases (marked with red triangles in Figure 1). No ARGs were found in the plasmids or inside mobile genetic elements, and no mutations in the *gyrA/parC/parE* loci conferring resistance to fluoroquinolone antibiotics were detected. No enterotoxins, cytotoxins, or hemolysin genes were found in the Q5 genome. However, the C41 strain carried a chromosomal *cdtABC* gene cluster encoding the cytolethal distending toxin (CDT). Several biosynthetic gene clusters (BGCs) of iron-chelating compounds (siderophores) were detected in both genomes. Enterobactin and yersiniabactin BGCs were found in the Q5 chromosome (marked with green and orange triangles in Figure 1). The enterobactin BGC was also detected in the C41 chromosome. The limited repertoire of ARGs, the absence of toxin-encoding genes, and the presence of siderophore biosynthesis genes make *E. coli* Q5 a promising probiotic candidate.

#### 2.1.3. Adhesion-Related Genes

Adhesion is the first step in the colonization of the intestine by microorganisms and is thus a required property for a probiotic strain. Adhesion-related genes were found in both sequenced genomes. The *E. coli* Q5 chromosome carries genes encoding the type 1 fimbriae (*fimB*, *fimE*, and *fimAICDFGH*), the *E. coli* common pilus (*ecpRABCDE*), curli (*csgDEFG* and *csgBAC*), and the FdeC adhesin (*fdeC*). The *E. coli* C41 chromosome contains gene clusters encoding the type 1 fimbriae (*fimB*, *fimE*, and *fimAICDFGH*), long polar fimbriae (*lpfABCD*), and curli (*csgDEFG* and *csgBAC*). The presence of detected adhesins should allow *E. coli* Q5 and C41 to attach to surfaces and colonize the intestine (see also below). Neither genome contains genes of fimbria associated with pyelonephritis (*pap*), fimbriae S and F1C (*sfa* and *foc*), afimbrial adhesins (*afa/dra*), or the *eae* gene, which codes for a protein required for the formation of attaching and effacing lesions.

#### 2.1.4. Bacteriocin Gene Clusters

The production of bacteriocins is believed to help probiotic strains compete with pathogenic strains for an ecological niche [5]. In order to evaluate the antimicrobial potential of the two strains, their genomes were screened with antiSMASH, PRISM4, and BAGEL4. *E. coli* Q5 genome contained three sets of genes required for production and export of (and self-immunity to) colicins Ia and Ib (both on the pQ501 plasmid) and colicin Y (on the pQ504 plasmid) (marked with blue triangles in Figure 1). An incomplete set of genes for microcin V production was found on the chromosome and in the pQ501 plasmid. Additionally, the *cbrA* gene responsible for resistance to colicin M was found on the Q5 chromosome (Table 1). The pC4102 plasmid contained a full set of functional genes needed for the production and export of pore-forming colicin E1 (*cea*, *cei*, *cel*). Additionally, the C41 chromosome contained the *cvpA* gene encoding microcin V production protein and the *cbrA* and *cbrC* genes conferring resistance to, respectively, colicins M and E2 (Table 1). The presence of complete gene sets for the production of different colicins supports the potential of the two strains (especially *E. coli* Q5) for development into probiotics.

### 2.2. In Vitro Analysis of E. coli Q5 and C41 Potential as Possible Probiotics

#### 2.2.1. Antimicrobial Activity in Spent Media

Spent media (cell-free supernatants) from *E. coli* Q5 and C41 cultures grown for 22 h were tested for their ability to inhibit the growth of various test strains. Such inhibition can be expected if supernatants contain bacteriocin produced during cultivation. Spent medium from *E. coli* M-17, a component of the commercial probiotic “Colibakterin”, was used as a control. *E. coli* Q5 and C41 supernatants inhibited, to various extent, the growth of avian pathogenic *E. coli* (BR4, BR35, BR37), diarrheagenic *E. coli* (CA29, CA43, CA46), *Klebsiella pneumoniae*, and *Staphylococcus aureus* (Table 2, Figure S1). *E. coli* O157 was modestly inhibited by the M-17 supernatant alone. *E. coli* Q5, but not other supernatants, inhibited the growth of *S. flexneri*. *E. coli* Q5 and C41 supernatants inhibited the growth of *E. coli* BR35 and CA46 more effectively than the M-17 control (*p* < 0.05). The C41 supernatant was also a better inhibitor of *Klebsiella pneumoniae* (*p* < 0.05). Neither supernatant affected on the growth of *Salmonella* Typhimurium, *Proteus mirabilis*, or *Pseudomonas aeruginosa.*

#### 2.2.2. Adhesion Ability

**Figure 2:**
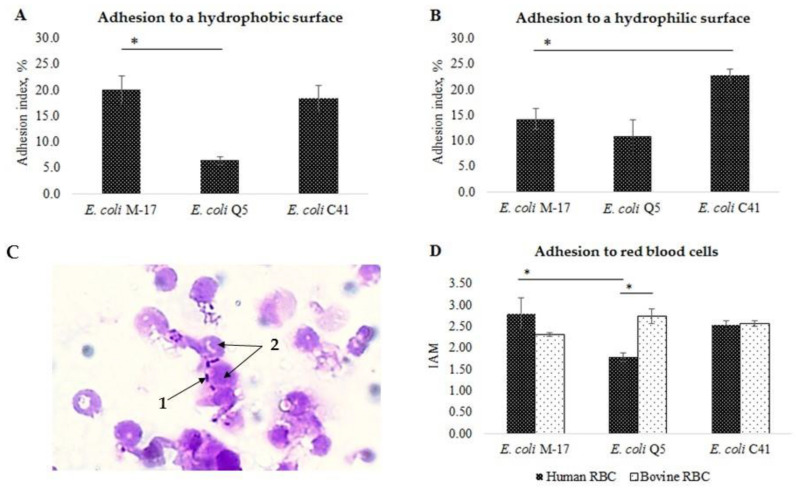
Adhesive properties of *E. coli* Q5, C41, and the control commercial probiotic strain M17. (**A**) Nonspecific adhesion to a hydrophobic surface (polystyrene). (**B**) Nonspecific adhesion to a hydrophilic surface (glass). (**C**) An example of specific adhesion of *E. coli* Q5, 30 min, staining with gentian violet, 1000×: 1—bacterial cells; 2—human red blood cells; (**D**) Level of specific adhesion. IAM—index adhesiveness of microorganisms. Columns—means; bars—mean deviations; «*» indicates a significant difference between levels of adhesion between strains (*t*-test, *p* < 0.05).

The level of nonspecific adhesion of *E. coli* Q5 to a hydrophilic surface was comparable, and in the case of adhesion to a hydrophobic surface, significantly lower than that of the control probiotic strain *E. coli* M-17 (*p* < 0.01). *E. coli* Q5 was low-adhesive to human red blood cells (RBC): the average adhesion index (AAI) was 0.83 ± 0.12, the adhesion coefficient (AC) was 0.47 ± 0.07, and the index adhesiveness of microorganisms (IAM) was 1.78 ± 0.09 (Figure 2). However, this strain was medium-adhesive to bovine RBC (AAI = 2.08 ± 0.18, AC = 0.76 ± 0.02, and IAM = 2.73 ± 0.16). *E. coli* C41 had a level of adhesion to a hydrophobic surface comparable to that of *E. coli* M-17. Adhesion to a hydrophilic surface was 22.8 ± 1.2%, which is significantly higher than that of the *E. coli* M-17 control (*p* < 0.01). *E. coli* C41 was classified as medium-adhesive and adhered well to both human RBC (AAI = 1.21 ± 0.04, AC = 0.48 ± 0.01, and IAM = 2.53 ± 0.09) and bovine RBC (AAI = 1.65 ± 0.08, AC = 0.65 ± 0.08, and IAM = 2.56 ± 0.05). It is worth noting that both strains, in contrast to *E. coli* M-17, had a greater affinity for bovine RBC, which could help to effectively colonize the intestines of animals.

#### 2.2.3. Antimicrobial Susceptibility and Lysogeny

The antimicrobial susceptibility of *E. coli* Q5 and C41 was analyzed by the disc diffusion method for a number of antibiotics, including ampicillin, cefoperazone, ceftriaxone, cefepime, aztreonam, meropenem, gentamicin, amikacin, norfloxacin, ciprofloxacin, levofloxacin, tetracycline, and chloramphenicol. Both strains were sensitive to every antibiotic tested.

Lysogeny is a potentially high-risk factor for a probiotic strain [20]. Ultraviolet (UV) irradiation of both strains for 70 s and 150 s, a condition that mobilizes prophages, did not lead to lysis, suggesting that *E. coli* Q5 and C41 strains are not lysogenic despite the presence of multiple integrated prophages (Table S2), which might be inactivated by mutations and/or are not mobilized in our experimental conditions.

### 2.3. In Vivo Analysis of Probiotic Properties of E. coli Q5 and C41

#### 2.3.1. The Effect of *E. coli* Q5 and C41 on the Physiological Parameters of Rats

Upon five-day oral administration of *E. coli* Q5 and C41 at daily doses of 5 × 10^8^ or 5 × 10^10^ colony-forming units (CFU)/per rat, the survival rate was 100%. There were no symptoms of disease or behavioral abnormalities; the animals were active. The average weight of rats fed with *E. coli* Q5 and C41 at a dose of 5 × 10^8^ CFU/rat·day exceeded the control by 2.7% and 0.5%, respectively (Table 3). At the higher dose of *E. coli* Q5 and C41, the growth-stimulating effect was lost, and the average body weight (BW) became lower than in the control group.

#### 2.3.2. Composition of Rat Intestinal Microbiota after Administration of *E. coli* Q5 and C41 and upon Experimental Infection with Toxigenic *E. coli* C55 after Preliminary Administration of *E. coli* Q5 and C41

**Figure 3:**
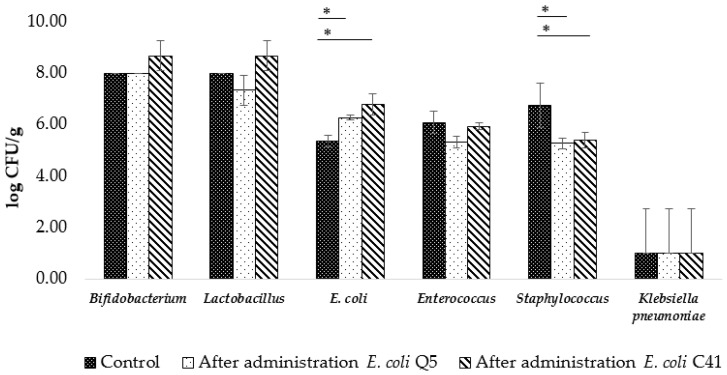
Changes in abundance of representative members of the rat intestinal microbiota after 5-day administration of *E. coli* Q5 or C41 (5 × 10^8^ CFU/rat·day). Columns—means; bars—mean deviations; «*» indicates a significant difference from control (intact rats) (*t*-test, *p* < 0.05).

The basic content of microorganisms in the intestinal microbiota of rats before the experiment was: 8.0 ± 0.0 lg CFU/g *Bifidobacterium* and *Lactobacillus*; 6.1 ± 0.2 lg CFU/g *Enterococcus;* 5.4 ± 0.2 lg CFU/g *E. coli*; 7.8 ± 0.1 lg CFU/g *Staphylococcus*; and 4.3 ± 0.3 lg CFU/g *Candida albicans*. When comparing the content of the intestinal microbiota of animals after the administration of *E. coli* Q5 and *E. coli* C41, there were no significant differences in the content of *Bifidobacterium, Lactobacillus,* and *Enterococcus*, whose content varied in the range of 8.0–8.7 lg CFU/g, 7.3–8.7 lg CFU/g, and 5.3–6.1 lg CFU/g, respectively (Figure 3). The content of *E. coli* significantly increased, from 5.4 ± 0.2 to 6.3 ± 0.1 lg CFU/g after administration of *E. coli* Q5 (*p* = 0.003) and from 5.4 ± 0.2 to 6.8 ± 0.4 lg CFU/g after the use of *E. coli* C41 (*p* = 0.002). Compared to the control, after simultaneous administration of both strains, there was a dramatic (average of two orders of magnitude) decrease in the number of *Staphylococcus* in the feces of rats.

**Figure 4:**
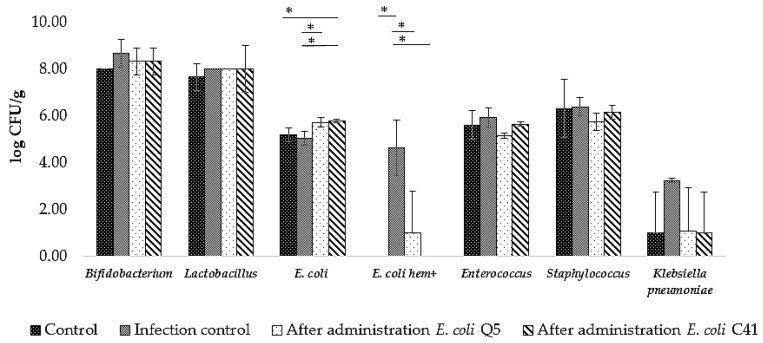
Changes in abundance of representative members of the rat intestinal microbiota after experimental infection with toxigenic strain *E. coli* C55 with or without preliminary 5-day administration of *E. coli* Q5 or C41 (5 × 10^8^ CFU/rat·day). Columns—means; bars—mean deviations; «*» indicates a significant difference from intact rats control or infection control (animals infected with *E. coli* C55) (*t*-test, *p* < 0.05).

As a result of toxigenic *E. coli* C55 infection, hemolytic *E. coli* (*E. coli* hem+) appeared in the intestinal microbiota of rats in the amount of 4.6 ± 1.2 lg CFU/g (Figure 4). The amount of *E. coli* hem+ was significantly lower in animals to which *E. coli* Q5 was administered (*p* = 0.04). After administration of *E. coli* C41, *E. coli* hem+ was not detected. It is important to emphasize that in the infection control group, the number of *C. albicans* significantly increased (*p* = 0.03) and the content of *K. pneumoniae* was on average 2.2 lg CFU/g higher than in the group of animals that received the probiotic strains prior to the introduction of toxigenic *E. coli* C55.

#### 2.3.3. Hematological and Biochemical Parameters of Rats after Administration of *E. coli* Q5 and C41 and during Experimental Infection with Toxigenic *E. coli* C55 after Preliminary Administration of *E. coli* Q5 and C41

The hematological and biochemical indices of rats are presented in Table 4. Red blood cell count (RBC), hemoglobin concentration ([Hb]), hematocrit (Ht), platelet count (PLT), white blood cell count (WBC), mean corpuscular volume (MCV), mean corpuscular hemoglobin (MCH), mean corpuscular hemoglobin concentration (MCHC), and glucose did not significantly differ from controls (*p* > 0.05) and were within the norm according to Wikivet [21]. After 5-day administration of *E. coli* Q5 or C41 at a dose of 5 × 10^8^ CFU/rat·day, the proportion of monocytes significantly increased (*p* < 0.05) but remained within the normal range of 0–5% [21]. Total protein and urea were normal (59–78 g/L and 3.07–7.28 μmol/L [22]) and did not show a significant difference (*p* > 0.05). The alanine aminotransferase (ALT) level decreased significantly after the introduction of probiotic bacteria but remained within the normal range of 35–80 U/L [23].

In the blood of rats in the control group infected with toxigenic *E. coli* C55, the concentration of alkaline phosphatase (ALP) increased 1.7 times but remained at the control level in animals that received *E. coli* Q5 or C41 prior to infection. In addition, the concentration of urea in the infection control group was slightly above the norm (3.07–7.28 μmol/L [22]).

#### 2.3.4. Histological Analysis of Small Intestine, Peyer’s Patches, Spleen, and Liver Morphology of Rats in Experimental Infection with Toxigenic *E. coli* C55 after Preliminary Administration of the *E. coli* Q5 and C41 Strains

**Figure 5:**
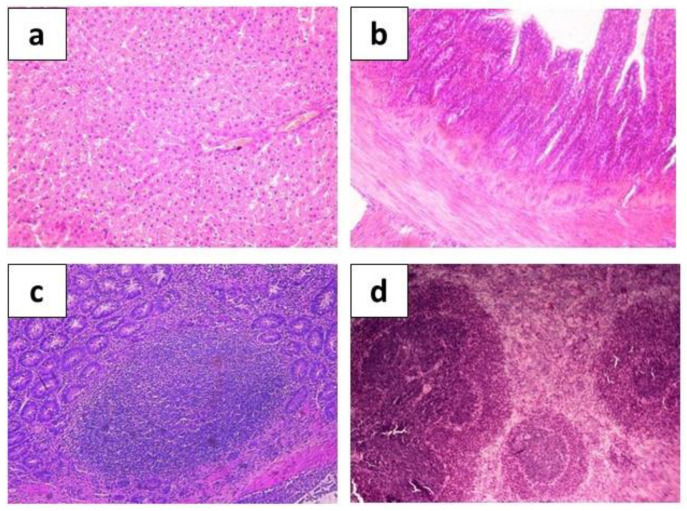
Hematoxylin-eosin-stained organ sections taken from control group animals show normal histological structures of rat hepatic tissue ((**a**), ×200), intestine ((**b**), ×200), Peyer’s patches ((**c**), ×400), and spleen ((**d**), ×200).

**Figure 6:**
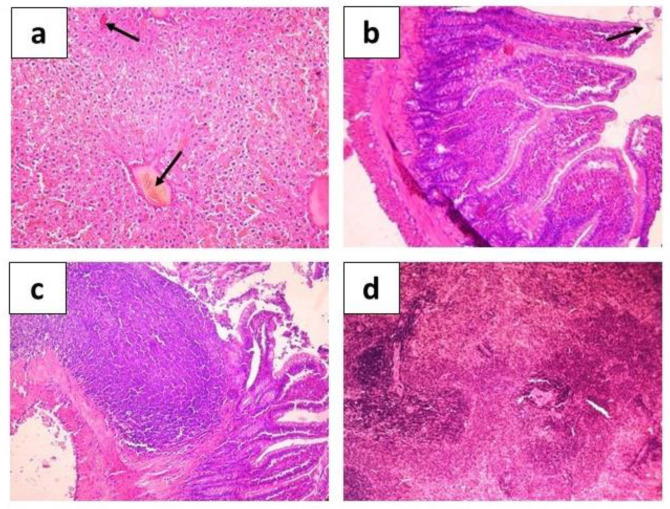
Hematoxylin-eosin stained organ sections of rats infected with *E. coli* C55 show histological structures of liver parenchyma with hemorrhage sites and congestion of blood vessels ((**a**), arrows, ×200), intestine with desquamation of the epithelium ((**b**), arrow, ×200) swelling of the stroma and the subepithelial part of the villi, Peyer’s patches ((**c**), ×400), and spleen ((**d**), ×200).

Administration of toxigenic *E. coli* C55 did not cause lethal effects but led to the appearance of distinct histopathological changes in the organs of rats in the infection control group compared to uninfected animals (Figure 5 and Figure 6). Lymphocytic cell infiltrates were found in the liver lobules. Hepatocytes showed degenerative changes: vascularization and dystrophic inflammation of liver cells were observed. Scattered areas of hemorrhage were recorded in the hepatic parenchyma of infected animals (Figure 6a). There was swelling of the stroma and the subepithelial part of the villi in the small intestine, and congestion of blood and lymphatic vessels was recorded in the mucosal and submucosal layers. There was an abundance of lymphocytes and granulocytes in the stroma of villi and crypts. Desquamation of the epithelium was observed on the surface of the mucous membrane of the small intestine (Figure 6b). In addition, activation of lymphoid tissue in Peyer’s patches, especially in the B-dependent zone, was detected compared with the control group (Figure 6c). In the colon, there was an increase in focal lymphocytic infiltration of the intestinal wall compared to the control-accumulations of lymphocytes are determined in the mucosa and submucosa, as well as in the muscular and serous layers (Figure S2). In the spleen, swollen stromal cells were determined in the red and white pulp. Lymphoid nodules of the white pulp were predominantly medium and small; most of them did not contain germinal centers (Figure 6d). There was also a decrease in the number and size of secondary follicles compared with the control group.

**Figure 7:**
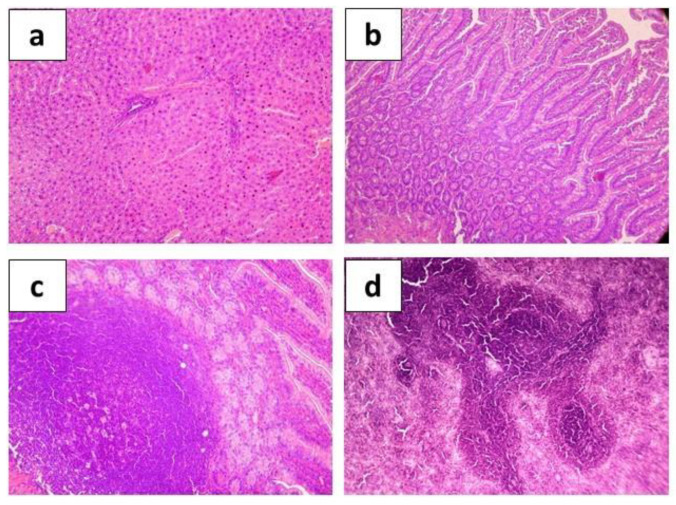
Hematoxylin-eosin stained organ sections taken from rats after experimental infection with toxigenic strain *E. coli* C55 after 5-day preliminary administration of *E. coli* Q5 (5 × 10^8^ CFU/rat·day) showing histological structure of hepatic tissue ((**a**), ×200), intestine ((**b**), ×200), Peyer’s patches ((**c**), ×400), and spleen ((**d**), ×200).

**Figure 8:**
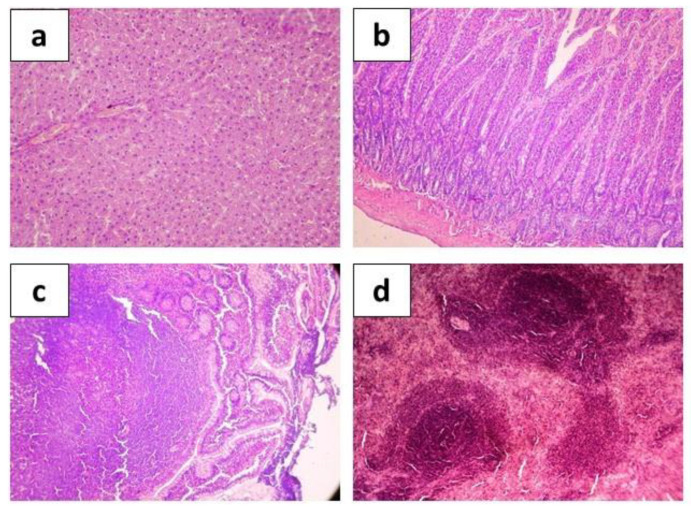
Hematoxylin-eosin stained organ sections taken from rats after experimental infection with toxigenic strain *E. coli* C55 after 5-day preliminary administration of *E. coli* C41 (5 × 10^8^ CFU/rat·day) showing histological structure of hepatic tissue ((**a**), ×200), intestine ((**b**), ×200), Peyer’s patches ((**c**), ×400), and spleen ((**d**), ×200).

Compared to infection control, a noticeable improvement in the state of organs was recorded in the group of rats infected after a preliminary 5-day administration of *E. coli* Q5 and C41 at a dose of 5 × 10^8^ CFU/rat·day. There were no infection-associated changes in the liver (Figure 7a and Figure 8a), and in the small intestine, epithelial cells formed an even monolayer without epithelial desquamation foci (Figure 7b and Figure 8b). Compared with the control group (intact rats), an increase in the number of active goblet cells and an increase in the mitotic activity of cells in the crypts were visually noted, and moderate diffuse lymphocyte infiltration of the mucosa was diagnosed. These data indicate the ability of probiotics to positively influence epithelial cell tight junction stability and intestinal goblet cell mucus production. Peyer’s patches were represented by clusters of large lymphoid nodules located in the mucosa and submucosa of the intestine (Figure 7c and Figure 8c). The nodules contained large germinal centers, occupying most of the follicle. In addition, there were many secondary lymphoid nodules containing germinal centers in the spleen (Figure 7d and Figure 8d). These data indicate antigenic stimulation of the host by probiotic bacteria, which in turn should stimulate intestinal immune cells, which contribute to the induction of mucosal immunity.

## 3. Discussion

Probiotics are living microorganisms that play an important role in maintaining overall health, strengthening the immune system, and preventing severe intestinal diseases in farm animals [6,24]. Significant progress has been made in the field of probiotics in recent decades; however, their mechanisms of action are still not fully understood. In this work, we describe and present two bacteriocin-producing *E. coli* strains that, based on the results of our analysis, hold promise for development as probiotics.

The production of bacteriocins is a key mechanism that allows probiotic *E. coli* to compete with pathogenic microorganisms in the intestine by inhibiting their growth [25]. Numerous studies have shown that *E. coli* bacteriocins are effective against diarrheagenic *E. coli* [12,13,15] and related enteropathogenic bacteria such as *Klebsiella*, *Salmonella*, and *Shigella* [26,27]. The antagonistic effect of commercial probiotic strain *E. coli* M-17 is due to the production of pore-forming colicin B and microcin V, which inhibit the synthesis of peptidoglycan by hydrolyzing lipid II [11]. The antagonistic properties of *E. coli* Nissle 1917 are due to the siderophores microcin M and microcin H47, which inhibit the ATP synthase [9,10]. For the *E. coli* Q5 strain studied in this work, antagonistic in vitro and in vivo activity is probably associated with the production of pore-forming colicins Ia, Ib, and Y since corresponding complete biosynthetic gene clusters have been found in its genome. In the case of *E. coli* C41, the production of pore-forming colicin E1 is the likely reason for antagonistic activity. Pore-forming bacteriocins bind to receptors of the Toll or Ton systems and become embedded in the lipid bilayer, leading to the formation of channels and leakage of cellular contents [8]. Both strains contain genes responsible for resistance to colicin M (and additionally to colicin E2 for *E. coli* C41), which should prevent their displacement by resident or pathogenic bacteriocin producers.

*E. coli* Q5 and C41 demonstrated in vitro antagonistic activity against most enteropathogens tested, including *E. coli* causing colibacillosis in farm animals. Oral administration of *E. coli* Q5 and C41 in vivo eliminated *S. aureus* and decreased *K. pneumoniae* titers during experimental infection with the enterotoxigenic *E. coli* C55. This is a very promising result given that *S. aureus* is considered the main causative agent of “contagious” mastitis in bovines [28], and *K. pneumoniae* is often associated with pneumonia and septicemia in foals [29].

Another anti-pathogenic mechanism of probiotic action is the binding and blocking of receptors in intestinal epithelial cells. The effectiveness of the interaction of microorganisms with surfaces depends on the expression of an extensive repertoire of genes encoding fimbrial and afimbrial adhesins [30,31]. The type 1 fimbriae encoded by *E. coli* Q5 and C41 attach in a mannose-dependent manner to eukaryotic cell receptors [31]. The *E. coli* Nissle 1917 probiotic strain also has type 1 fimbriae and is curly [10]. Enteropathogenic *E. coli* uses type 1 fimbriae to attach to intestinal epithelial cells. Thus, probiotic strains exclude the binding of pathogens by attaching to the same receptors. Adhesive amyloids (curly), encoded by both strains, are involved in adhesion to surfaces [32]. The FdeC adhesin encoded by *E. coli* Q5 has a high affinity for epithelial cells and provides protection against urinary tract infections [33].

Strains with good adhesive ability colonize the intestine better [34]. Yet, high-adhesive strains are not considered promising for probiotic development, as they displace not only pathogenic but also autochthonous microorganisms [35]. Therefore, most of the probiotic strains used are low- or medium–adhesive [10]. In vitro studies have shown that both strains were medium-adhesive and had a greater affinity for bovine RBC than *E. coli* M-17, which may allow *E. coli* Q5 and C41 to effectively colonize the intestines of animals.

A crucial property for the practical application of probiotic strains is biosafety. Functional annotation of the *E. coli* Q5 genome allowed us to confirm the absence of enterotoxin genes, hemolysins, virulence-associated fimbriae (such as *pap*, *sfa*, *afa/dra* operons), and mobile ARGs. The *E. coli* C41 chromosome encodes the Cdt toxin, a pathogenicity factor. However, there was no toxic effect when *E. coli* C41 was administered at a dose of 5 × 10^10^ CFU/rat·day. The presence of *cdtABC* is a risk factor that; however, can be eliminated by removing the gene. Another risk factor is the presence of several prophage elements in the genomes of *E. coli* Q5 and C41. However, our in vitro studies indicated the apparent absence of lysogenic activity in both strains.

The two strains had no negative effect on the physiological (the body weight gain was within the norm) or hematological parameters of the rats. The total proteins, ALP, and ALT were normal, which confirms the absence of hepatotoxicity in the strains [24,36]. In addition, the administration of *E. coli* Q5 and C41 countered the increase in ALT levels during experimental infection with a toxigenic *E. coli*. According to Shahverdi et al., this effect is characteristic of probiotic strains and indicates their hepatoprotective role [36]. Maintaining the levels of urea and creatinine in the normal range indicates the absence of any kidney disorders in rats. Finally, administration of both strains did not lead to significant changes in the composition of the native microbiota; the level of beneficial representatives such as *Bifidobacterium*, *Lactobacillus*, and *Enterococcus* remained unchanged.

Stabilization and maintenance of the integrity of the intestinal barrier are mechanisms of probiotic action that provide protection against pathogens and the toxins they produce. The most severe animal diseases are caused by enterohemorrhagic (producing shiga-toxin Stx1 and/or Stx2 that stop protein synthesis in endothelial target cells) and enterotoxigenic (producing enterotoxin EAST1 and/or enterohemolisin EhxA) *E. coli* strains [15,37,38,39]. These toxins, produced by beta-lactam-resistant *E. coli* C55, lead to pathological changes in the intestines of infected rats (inflammation, epithelial desquamation, focal lymphocytic infiltration), and increased ALP levels. We show that preliminary administration of bacteriocin-producing *E. coli* for 5 days at a dose of 5 × 10^8^ CFU/rat·day protected rats from colonization and pathogenic effects of *E. coli* C55. If the proportion of *E. coli* hem+ in the infection control group was more than half of all *Escherichia*, then after the preliminary administration of *E. coli* Q5, *E. coli* hem+ were detected in only one animal, and after the administration of *E. coli* C41, *E. coli* hem+ were not detected at all. Pre-emptive oral administration of our strains prevented the destruction of the intestinal barrier (there was no epithelial desquamation or inflammation), presumably by blocking the access of *E. coli* C55 and its metabolites to subepithelial cells. In a mouse model, the introduction of *E. coli* Nissle 1917 protected the intestinal barrier from dysfunction due to a more pronounced expression of the tight junction molecules regulating intestinal permeability (Ukena et al., 2007). The mechanism(s) of intestinal barrier protection operational in the cases of *E. coli* C41 and Q5 remain to be determined.

In summary, positive effects of bacteriocin-producing *E. coli* are associated with inhibition of enteropathogens through bacteriocin production, competition for adhesion sites, improving the balance of the natural intestinal microbiota, and maintaining the integrity of the epithelial barrier by stimulating the secretion of mucin glycoproteins, antimicrobial proteins, tight junction molecules, modulation of metabolic and immune processes, and likely other mechanisms. Thus, our work demonstrated that short-term oral administration of *E. coli* Q5 and C41 to rats contributed to the preservation of intestinal homeostasis and provided protection from external influences, including infection with an enterotoxigenic beta-lactam-resistant *E. coli* strain. Given all the evidence, these two strains are promising candidates for development as probiotics for farm animals.

## 4. Materials and Methods

### 4.1. Bacterial Strains

Earlier, we studied 97 *E. coli* isolates obtained from fecal samples of healthy farm animals from industrial and private farms in Russia [18]. As a result of the study, two bacteriocin-producing strains were selected: *E. coli* Q5 was obtained from a healthy quail and *E. coli* C41 from a healthy cow. These strains presumably had high probiotic potential. *E. coli* Q5 and *E. coli* C41 strains were deposited in the All-Russian Collection of Microorganisms (VKM) under the numbers B-3706D and B-3707D, respectively. A toxigenic strain of *E. coli* C55 was isolated from a calf with diarrhea. *E. coli* C55 produced intestinal toxins (Stx1, East1, and EhxA) and was resistant to beta-lactam antibiotics (ampicillin, ceftriaxone, cefepime, and cefoperazone). This strain was used in the current work to simulate experimental toxicoinfection. The characteristics of all strains used in this work are presented in Table 5.

### 4.2. Genome Sequencing and Assembly

*E. coli* Q5 and *E. coli* C41 genomic DNA was extracted from the overnight cultures grown at 37 °C using the GeneJET Genomic DNA purification kit (Thermo Scientific, Vilnius, Lithuania). DNA was sequenced using Oxford Nanopore Technologies (ONT). Sequencing libraries were prepared from the non-sheared DNA using the Native Barcoding kit (SQK-NBD114-24; ONT, Oxford, UK) with enrichment of long fragments using the Long Fragment Buffer (LFB) according to the manufacturer’s protocol. Sequencing was performed on MinION using the R10.4.1 flow cell (FLO-MIN114; ONT, Oxford, UK) with a translocation rate of 400 bps. Basecalling was performed using Guppy 6.0.1 [40] in the “hac” mode. Default parameters were used for all software unless otherwise specified. Draft genomes were assembled with Flye (v 2.9.1) [41]. The assembly was subsequently polished with medaka (v 1.7.2) using ONT reads, and assembly graphs were manually inspected in Bandage (v 0.8.1) [42].

### 4.3. Genome Annotation and Analysis

Polyshed genome assemblies containing circular replicons were further annotated using PGAP (v 6.1) [43]. Virulence-associated genes (VAGs) were detected using VirulenceFinder (v 2.0) [44] and VRprofile2 [45]. Antibiotic-resistance genes (ARGs) were predicted with Abricate [46] using the NCBI AMRFinderPlus database [47]. Mutations conferring antibiotic resistance were searched using ResFinder (v 4.1) [44]. Biosynthetic gene clusters (BGC) and bacteriocins were predicted using antiSMASH (v 7.0) in a “loose” mode [48,49], PRISM4 [50], and BAGEL4 [51]. Prophages were predicted with PHASTEST [52]. Plasmid incompatibility groups were predicted with PlasmidFinder-2.0 [53].

### 4.4. Data Deposition

Raw reads for *E. coli* C41 and *E. coli* Q5 whole-genome sequencing were deposited in the Sequence Read Archive (SRA) under SRR24834172 and SRR24834173 accessions, respectively. Annotated genome assemblies obtained in this study were deposited in the NCBI BioProject PRJNA980458, GenBank accession numbers CP127252-CP127254 (*E. coli* C41) and CP127255-CP127260 (*E. coli* Q5).

### 4.5. Antimicrobial Activity of Cell-Free Supernatants of E. coli Strains

The in vitro antagonistic effect of probiotic *E. coli* was assessed by evaluating the bacterial growth of test-strains (Table 5) in the presence of cell-free supernatants of the studied *E. coli* strains in the culture medium. *E. coli* M-17 was obtained from the probiotic “Colibakterin” (MICROGEN NPO JSC, Nizhniy Novgorod, Russia) and used as a control strain. *E. coli* Q5, *E. coli* C41, and *E. coli* M-17 strains were overnight cultured in liquid Luria-Bertani medium (LB medium, “Difco”, Le Pont de Claix, France) at 37 °C without aeration. The grown bacterial cultures were transferred into Eppendorf tubes and centrifuged for 10 min at 13,000 rpm. The supernatants were sterilized using Millex^®^-GS membrane filters (“Merck Milli-pore Ltd.”, Carrigtwohill, Ireland) with a pore diameter of 0.22 μm. Supernatants were stored at −20 °C. Suspensions of 24 h cultures of the test-strains diluted to a concentration of 10^6^ CFU/mL and cell-free supernatants of probiotic strains were introduced into the wells of the 96-well microtiter plates in a ratio of 1:1 and incubated at 37 °C for 24 h without shaking. Subsequently, the optical density OD_600_ of cultures was measured using the plate reader INFINITE M1000 (Tecan Austria GmbH, Grödig, Austria), and the percentage of growth inhibition after 22 h of co-cultivation was calculated, taking as 100% the optical density of the culture grown in the control wells.

### 4.6. Nonspecific Adhesion of E. coli Strains

The study of bacterial nonspecific adhesion was carried out in glass penicillin vials (hydrophilic surface) and in polystyrene 96-well plates (Medpolimer, Saint Petersburg, Russia) (hydrophobic surface), according to Nikolaev Yu.A. [54]. Bacterial cells were deposited at 8000 rpm, washed twice in a phosphate buffer, standardized to 0.150–0.200 OD_540_ units, and 3.0 mL were injected into vials and 200 mL into the wells of the microplate. Vials and plates were placed for 1 h in a thermostat at 37 °C with stirring at 150 rpm. The adhesion index was understood as the number of cells adhering to the walls of the vial/plates, expressed in % of their initial number, and was calculated as follows:

$$Adhesion\;index=\left(1-\frac{{OD}_{final}}{{OD}_{initial}}\right)\cdot 100\%$$

where ${OD}_{initial}$ and ${OD}_{final}$ are the optical densities at the initial moment of time and after 1 h, respectively.

### 4.7. Specific Adhesion of E. coli Strains

The study of bacterial specific adhesion to red blood cells was carried out according to the Brillis method in Eppendorf tubes [55]. To account for the adhesive properties of bacteria, human red blood cells O (I) of the Rh (+) blood group were used (“Biomed”, a branch of FSUE “Microgen”, Perm, Russia). Erythrocytes contain glycophorin on their surface, which is identical to the glycocalyx of epithelial cells [56]. Erythrocytes were washed in saline phosphate buffer (PBS), then diluted to 10^8^ cells/mL. The bacteria were grown overnight, washed with phosphate buffer, and a suspension was prepared at a concentration of 10^8^ cells/mL. Then a bacterial suspension was mixed with erythrocyte mass in a ratio of 1:1 and incubated at 37 °C with stirring at 120 rpm for 30 min. Blood smears were prepared and stained with a 0.5% solution of gentian violet [57]. During optical microscopy of the preparations, the following indicators were taken into account: average adhesion index (AAI), which is the average number of microorganisms attached to the surface of a single red blood cell; and adhesion coefficient (AC), the percentage of red blood cells having bacteria on the surface. The index adhesiveness of microorganisms (IAM) was calculated as follows:

$$IAM=\frac{AAI}{AC}$$

Counting was carried out on 100 cells, looking through the entire glass slide. Depending on the IAM values, microorganisms were considered non-adhesive (IAM < 1.75), low-adhesive (IAM = 1.76–2.49), medium-adhesive (IAM = 2.50–3.99), and highly adhesive (IAM > 4.0).

### 4.8. Antimicrobial Susceptibility

The strains were tested by the disk-diffusion method using Muller-Hinton agar (“FBIS SRCAMB”, Obolensk, Russia) and disks (“NICF”, St. Petersburg, Russia) for sensitivity to ampicillin (10 µg), cefoperazone (75 µg), ceftriaxone (30 µg), cefepime (30 µg), meropenem (10 µg), aztreonam (30 µg), amikacin (30 µg), gentamicin (10 µg), ciprofloxacin (5 µg), levofloxacin (5 µg); norfloxacin (10 µg), tetracycline (30 µg), chloramphenicol (30 µg). The determination of the sensitivity of *E. coli* strains to antibiotics was carried out in accordance with the clinical guidelines “Determination of the sensitivity of microorganisms to antimicrobial drugs” of the Interregional Association for Clinical Microbiology and Antimicrobial Chemotherapy (IACMAC, Version-2018-03).

### 4.9. Bacteriophage Induction

Bacterial overnight cultures were diluted in PBS in order to obtain a concentration of 1 × 10^5^ to 1 × 10^6^ bacteria per ml, and 20 mL of such diluted overnight cultures were transferred into standard Petri dishes for exposure to the continuous UV-light treatment (260 nm) for 70 s or 150 s. After the UV exposure, the cultures were incubated for 1 h at 37 °C and then mixed with a culture of the sensitive strain *E. coli* DH5a and added to melted 0.6% agar (46 °C), mixed, and poured onto LB agar plates. After a 24 h incubation at 37 °C the presence of lysis zones in the sensitive strain was screened for.

### 4.10. Probiotic and Pathogenic Inocula Preparation

To prepare a probiotic suspension, *E. coli* Q5 and C41 were grown in LB broth for 24 h at 37 °C without aeration. Then the suspensions of microorganisms were centrifuged at 5000 rpm for 10 min, the supernatant was removed, and the sediment was resuspended in saline. The OD was measured and brought to the final concentration of 5 × 10^8^ or 5 × 10^10^ CFU/mL. The pathogenic inoculum of *E. coli* C55 at a concentration of 5 × 10^8^ CFU/mL was prepared in a similar way. The suspensions were stored in vials at a temperature of 4 °C and used for administration to rats.

### 4.11. Experimental Design In Vivo

Forty-eight 180 day-old white male rats of the Wistar line were used for in vivo experiments. Experiments on rats were conducted following guidelines set by the Ethics Committee. General animal care was carried out in accordance with State Standard No. 33215-2014, “Guidelines for accommodation and care of animals. Environment, housing and management” [58]. The rats were caged in the animal house, where the temperature ranged from 23 °C to 26 °C. The animals received free access to feed (standard pellets) and drinking water (ad libitum) during all experiments. Three rats were used to analyze background hematological and biochemical parameters and the composition of the intestinal microbiota before the experiment. The remaining rats were randomly divided into six groups. The design of the in vivo experiment is presented in Table 6.

The first group (control) included intact animals (*n* = 10) that received 1 mL of saline throughout the experiment.

The second group (infection control) included rats (*n* = 5) that received 1 mL of saline for 5 days, then per animal once orally infected with the toxigenic *E. coli* C55 (5 × 10^8^ CFU suspended in 1 mL of saline).

The third (*n* = 10) and fourth (*n* = 10) groups included rats that received *E. coli* Q5 or *E. coli* C41, respectively, orally (5 × 10^8^ CFU suspended in 1 mL of saline), daily for 5 days, with drinking water. Then, after administration of probiotic bacteria, five rats from each group were removed for analysis of hematological and biochemical parameters. The remaining animals were infected with *E. coli* C55 (5 × 10^8^ CFU suspended in 1 mL of saline) orally with water per animal. After 3 days after infection, all rats were euthanized, and the blood and organs of the rats were taken for analysis.

The fifth (*n* = 5) and sixth (*n* = 5) groups included rats that received *E. coli* Q5 or *E. coli* C41, respectively, orally (5 × 10^8^ CFU suspended in 1 mL of saline) daily for 5 days with drinking water. The body weight (BW) of rats in the first, second, third, and fourth groups was measured before the experiment, after administration of probiotic bacteria, and after infection with *E. coli* C55. The BW of animals in the fifth and sixth groups was measured before the experiment and after taking probiotic microorganisms. Throughout the study, the behavior and appearance of animals, water consumption, and food consumption were monitored to determine whether there were any deviations from normal behavior.

### 4.12. Analysis of the Composition of the Intestinal Microbiota

The feces of randomly chosen rats from each group were used as material for bacteriological analysis. The bacteriological analysis of the microbial intestinal community was performed by direct plating (colony-forming unit count, CFU) on selective solid media: Pseudomonas CN Agar (Laboratorios Conda S.A., Madrid, Spain), Endo Agar for *E. coli*, Ploskireva Agar for Proteus, Egg-salt Agar for Staphylococcus, Blaurocca medium for Bifidobacteria, MRS Agar for Lactobacillus, Iron Sulfite Modified Agar №3 for Clostridium, and Sabouraud Agar №2 for Candida (“FBIS SRCAMB”, Obolensk, Russia). After infection, feces were inoculated on blood agar with ampicillin. Ampicillin-resistant colonies with hemolysis representing an experimental *E. coli* C55 infection were counted. The obtained CFU were recalculated to 1 g of the chyme content.

### 4.13. Hematological and Biochemical Blood Analysis

Blood samples were taken directly from the heart using a syringe. Analysis of red blood cell count (RBC), hemoglobin concentration ([Hb]), hematocrit (Ht), platelet count (PLT), and white blood cell count (WBC) was performed using the automated Hematological Analyzer (MINDRAY BS-3600, Shenzhen, China). Using RBC, Ht, and [Hb], the average corpuscular volume (MCV), average corpuscular hemoglobin (MCH), and average concentration of corpuscular hemoglobin (MCHC) were calculated according to standard formulas [59].

Blood samples were centrifuged at 1000 RPM for 10 min (Eppendorf 5415R, Germany) and analyzed for the following serum biochemical parameters: glucose, total protein, creatinine, urea, and levels of enzymes phosphatase (ALP) and alanine aminotransferase (ALT) using a Biochemical Analyzer (MINDRAY BS-200, Shenzhen, China).

### 4.14. Histologic Analysis

Samples of intestine, Peyer’s patches, spleen, and liver from rats were fixed in 10% neutral formalin in phosphate buffer (pH 7.2) and poured into “Histomix” paraffin (BioVitrum, Saint Petersburg, Russia). The paraffin sections were stained with hematoxylin (BioVitrum, Russia) and eosin (BioVitrum, Saint Petersburg, Russia) to evaluate tissue morphology under a light microscope (Olympus, Tokyo, Japan).

### 4.15. Statistical Analysis

The data were presented as the arithmetic mean and its mean deviation (M ± m). Statistical analysis was performed using the Student’s *t*-test in STATISTICA 10.0. A *p*-value of less than 0.05 was considered significant.

## 5. Conclusions

This study presents a comprehensive assessment of the probiotic characteristics of two bacteriocin-producing strains (*E. coli* Q5 and C41) using in silico, in vitro, and in vivo approaches. The results demonstrate that oral administration of *E. coli* Q5 and C41 to rats did not cause side effects or signs of clinical disease but contributed to the preservation of intestinal homeostasis and had a preventive effect by protecting against the pathogenic effects of a toxigenic *E. coli* strain. Given that maintaining effective symbiosis between the host organism and the intestinal microbiota is currently considered a necessary component of the veterinary strategy to ensure animal health, our results form the basis for research and development of a probiotic based on the studied strains to be used for the treatment and prevention of infectious diseases in farm animals.

## Figures and Tables

**Table 1:** Description of bacteriocin-related genes found in the *E. coli* Q5 and C41 genomes.

Strain	Colicin	Genes Found
*E. coli* Q5	colicin Ia *	*cia* (QQ972_24345, pQ501), *iia* (QQ972_24350, pQ501)
	colicin Ib *	*cib* (QQ972_24045, pQ501), *iib* (QQ972_24050, pQ501)
	colicin Y *	*crl*, *cui*, *cya* (pQ504)
	microcin V	*cvaC* (frameshifted, pQ501), *cvi* (*cvi* and QQ972_24735, pQ501), *cvpA* (chr)
	colicin M	*cbrA* (chr)
*E. coli* C41	colicin E1 *	*cei* (QQ971_24605), *cea*, *cel* (QQ971_24650) (all pC4102)
	microcin V	*cvpA* (chr)
	colicin M	*cbrA* (chr)
	colicin E2	*cbrC* (chr)

Note. «*»—indicates a full set of functional genes required for bacteriocin production, processing, and export.

**Table 2:** Antagonistic activity of cell-free supernatants of *E. coli* M-17, Q5, and C41 against test-strains, M ± m.

Test-Strain	Growth Inhibition Index of Test-Strains after 22 h of Cultivation, %		
	*E. coli* M-17	*E. coli* Q5	*E. coli* C41
*E. coli* BR4	42.1 ± 9.5	38.3 ± 7.9	46.9 ± 13.3
*E. coli* BR35	31.9 ± 8.2	46.6 ± 5.4 *	55.9 ± 5.7 *
*E. coli* BR37	25.7 ± 11.3	41.3 ± 5.6	43.2 ± 2.6
*E. coli* CA29	38.4 ± 5.3	21.0 ± 3.5	22.4 ± 4.6
*E. coli* CA43	32.4 ± 5.2	24.6 ± 2.6	33.9 ± 4.5
*E. coli* CA46	10.2 ± 1.5	31.3 ± 2.8 *	37.0 ± 4.3 *
*E. coli* O157	12.6 ± 9.7	0	0
*K. pneumoniae*	35.6 ± 4.0	25.8 ± 0.9	55.1 ± 2.5 *
*S. aureus*	18.8 ± 7.3	15.2 ± 2.8	14.2 ± 3.8
*S. flexneri*	0	25.6 ± 11.4 *	0
*S.* Typhimurium	0	0	0
*P. mirabilis*	0	0	0
*P. aeruginosa*	0	0	0

Note. «*»—significantly more than *E. coli* M-17 (*t*-test, *p* < 0.05).

**Table 3:** Measured physiological parameters of the rats, M ± m.

Parameter	Control	*E. coli* Q5, CFU/Rat·Day		*E. coli* C41, CFU/Rat·Day	
		5 × 10^8^	5 × 10^10^	5 × 10^8^	5 × 10^10^
Survival rate, %	100	100	100	100	100
BW before administration, g	372 ± 35	358 ± 14	372 ± 26	349 ± 28	358 ± 14
BW after administration, g	392 ± 39	387 ± 22	371 ± 28	369 ± 27	372 ± 16
BW gain, g	20 ± 5	29 ± 13	−1 ± 5	20 ± 8	14 ± 6
BW gain, %	5.3 ± 1.1	8.0 ± 4.6	−0.3 ± 1.4 *	5.8 ± 2.4	4.0 ± 4.3

Note. BW—body weight. «*»—significant difference from the control (intact rats) (*t*-test, *p* < 0.05).

**Table 4:** Hematological and biochemical indices of the rats, M ± m.

Parameter	Probiotic Administration (5 Days)			Experimental Infection *E. coli* C55 (8 Days)			
	Control	*E. coli* Q5	*E. coli* C41	Control	Infection Control	After Administration *E. coli* Q5	After Administration *E. coli* C41
**Hematological parameters**							
RBC, ×10^12^/L	8.8 ± 0.4	8.7 ± 0.2	9.3 ± 0.1	8.7 ± 0.3	8.9 ± 0.2	8.6 ± 0.4	8.9 ± 0.2
Hb, g/L	153 ± 4	151 ± 7	161 ± 4	152 ± 6	158 ± 4	149 ± 6	158 ± 3
Ht, %	45 ± 1	45 ± 2	48 ± 1 *	45 ± 2	47 ± 1	44 ± 2	47 ± 1
PLT, ×10^9^/L	760 ± 94	653 ± 34	673 ± 80	839 ± 112	620 ± 94	739 ± 39	770 ± 75
WBC, ×10^9^/L	13.0 ± 1.0	13.2 ± 1.6	10.3 ± 2.4	12.5 ± 3.2	11.1 ± 2.0	12.5 ± 1.8	12.1 ± 1.5
Neutrophils, %	26 ± 7	38 ± 7	29 ± 6	37 ± 11	38 ± 8	29 ± 9	36 ± 7
Lymphocytes, %	71 ± 8	55 ± 9	67 ± 6	65 ± 7	58 ± 7	54 ± 19	60 ± 7
Eosinophils, %	2 ± 2	2 ± 2	1 ± 1	2 ± 2	1 ± 1	2 ± 1	1 ± 1
Basophils, %	0	0	0	0	0	0	0
Monocytes, %	1 ± 0	5 ± 2 *	3 ± 1 *	2 ± 1	3 ± 2	4 ± 2	2 ± 1
MCV, fL	51.5 ± 1.3	51.8 ± 0.6	41.5 ± 16.6	51.4 ± 1.4	52.9 ± 2.1	51.4 ± 0.5	52.6 ± 1.1
MCH, pg	17.6 ± 0.4	17.4 ± 0.3	17.4 ± 0.3	17.5 ± 0.3	17.8 ± 0.5	17.4 ± 0.1	17.7 ± 0.2
MCHC, %	34.1 ± 0.1	33.7 ± 0.3	33.8 ± 0.2	34.0 ± 0.3	33.7 ± 0.5	33.8 ± 0.3	33.7 ± 0.4
**Biochemical parameters**							
Glucose, mmol/L	6.8 ± 0.3	6.5 ± 0.5	7.1 ± 0.4	6.5 ± 0.3	6.6 ± 0.4	6.7 ± 0.4	7.1 ± 0.5
Total protein, g/L	67.6 ± 1.7	67.1 ± 1.9	66.3 ± 2.1	73.1 ± 2.9	71.2 ± 2.8	68.9 ± 2.1	69.0 ± 2.2
Creatinine, μmol/L	64.2 ± 1.0	59.2 ± 3.4 *	55.8 ± 3.8 *	61.8 ± 6.8	54.4 ± 4.2	45.9 ± 4.7 #	47.2 ± 4.2 #
Urea, μmol/L	7.1 ± 0.6	7.2 ± 0.3	7.1 ± 0.8	6.8 ± 0.7	7.6 ± 0.6	7.2 ± 0.5	6.8 ± 0.5
ALP, U/L	475 ± 135	539 ± 117	604 ± 101	521 ± 69	921 ± 79 #	569 ± 73 ^α^	602 ± 43 ^α^
ALT, U/L	85.2 ± 9.0	69.3 ± 7.8	60.8 ± 3.9 *	90.6 ± 7.9	93.0 ± 7.6	92.3 ± 8.4	75.9 ± 4.8 #^α^

Note. RBC—red blood cell, Hb—hemoglobin, Ht—hematocrit, PLT—platelet count, WBC—total leukocyte count, PI—phagocytic index, ALP—alkaline phosphatase, ALT—alanine aminotransferase, MCV—mean corpuscular volume, MCH—mean corpuscular hemoglobin, MCHC—mean corpuscular Hb concentration. «*» or «#»—significant difference from the control (intact rats, 5 days) and control (intact rats, 8 days), respectively (*t*-test, *p* < 0.05), α—significant difference from the infection control group in which animals were infected with *E. coli* C55 (*t*-test, *p* < 0.05).

**Table 5:** Bacterial strains used in this work.

Strain	Collection/Source	Collection Number
Studied bacteriocin-producing strains		
*Escherichia coli* Q5	VKM/Feces of healthy quail from industrial farms, Perm, Russia	B-3706D
*Escherichia coli* C41	VKM/Feces of healthy cattle from industrial farms, Perm, Russia	B-3707D
Test-strains used for the antagonistic activity experiment		
*Escherichia coli* BR4	“Ex culture collection”, University of Ljubljana, Slovenia	L-5838
*Escherichia coli* BR35		L-5865
*Escherichia coli* BR37		L-5868
*Escherichia coli* CA29	Feces of cattle from industrial farms, Perm, Russia	-
*Escherichia coli* CA43		-
*Escherichia coli* CA46		-
*Escherichia coli* O157	State collection of pathogenic microorganisms and cell cultures (SCPM), Obolensk, Russia	240329
*Klebsiella pneumoniae* subsp. *pneumoniae* ATCC 700603		B-7474
*Proteus mirabilis* №H-237		160120
*Pseudomonas aeruginosa* ATCC27853		41501
*Staphylococcus aureus* ATCC 6538 (FDA 209P)		201108
*Shigella flexneri* №170		232151
*Salmonella enterica* serovar Typhimurium №1135	Feces of a patient with acute enteritis in the medical facility, Perm, Russia	-
Control strain		
*Escherichia coli* M-17	“Colibakterin”	-
Strain used to simulate experimental infection		
*Escherichia coli* C55	Feces of cattle from industrial farms, Perm, Russia	-

**Table 6:** In vivo experiment design.

Time Period, Days	Number of Euthanized Rats						Action
	Control (10 in Total)	Infection Control (5 in Total)	*E. coli* Q5, 5 × 10^8^ CFU (10 in Total)	*E. coli* C41, 5 × 10^8^ CFU (10 in Total)	*E. coli* Q5, 5 × 10^10^ CFU (5 in Total)	*E. coli* C41, 5 × 10^10^ CFU (5 in Total)	
0							Body mass measurement
1–4							Administration probiotic bacteria at a dose 5 × 10^8^ or 5 × 10^10^ CFU
5	−5		−5	−5	−5	−5	Body mass measurement, analysis of microbiota composition, hematological and biochemical parameters, infection with a toxigenic *E. coli* C55
6–7							Monitoring the condition of rats
8	−5	−5	−5	−5			Body mass measurement, analysis of microbiota composition, hematological and biochemical parameters, organ removal *

Note. «*»—organs for histologic analysis were removed from two randomly selected rats.

## Data Availability

Data are contained within the article or Supplementary Materials.

## Supplementary Materials

The supporting information can be downloaded at: https://www.mdpi.com/article/10.3390/ijms241612636/s1.

## References

[1] Tomley F.M., Shirley M.W. (2009). Livestock infectious diseases and zoonoses. Philos. Trans. R. Soc. Lond. B Biol. Sci..

[2] European Food Safety Authority (EFSA), European Centre for Disease prevention and Control (ECDC) (2021). The European Union Summary Report on Antimicrobial Resistance in zoonotic and indicator bacteria from humans, animals and food in 2018/2019. EFSA J..

[3] Okello E., Williams D.R., ElAshmawy W.R., Adams J., Pereira R.V., Lehenbauer T.W., Aly S.S. (2021). Survey on antimicrobial drug use practices in California preweaned dairy calves. Front. Vet. Sci..

[4] Parker J.K., Davies B.W. (2022). Microcins reveal natural mechanisms of bacterial manipulation to inform therapeutic development. Microbiology.

[5] Das T.K., Pradhan S., Chakrabarti S., Mondal K.C., Ghosh K. (2022). Current status of probiotic and related health benefits. App. Food Res..

[6] Hossain M.I., Sadekuzzaman M., Ha S.D. (2017). Probiotics as potential alternative biocontrol agents in the agriculture and food industries: A review. Food Res. Int..

[7] Mazurek-Popczyk J., Pisarska J., Bok E., Baldy-Chudzik K. (2020). Antibacterial activity of bacteriocinogenic commensal *Escherichia coli* against zoonotic strains resistant and sensitive to antibiotics. Antibiotics.

[8] Rebuffat S., Drider D., Rebuffat S. (2011). Bacteriocins from gram-negative bacteria: A classification?. Prokaryotic Antimicrobial Peptides.

[9] Grozdanov L., Raasch C., Schulze J., Sonnenborn U., Gottschalk G., Hacker J., Dobrindt U. (2004). Analysis of the genome structure of the nonpathogenic probiotic *Escherichia coli* strain Nissle 1917. J. Bacteriol..

[10] Sonnenborn U., Schulze J. (2009). The non-pathogenic *Escherichia coli* strain Nissle 1917—features of a versatile probiotic. Microb. Ecol. Health Dis..

[11] Belova I.V., Tochilina A.G., Solovieva I.V., Gorlova I.S., Efimov E.I., Zhirnov V.A., Ivanova T.P. (2017). Phenotypic and genotypic characteristics of the probiotic strain *E. coli* M-17. Mod. Probl. Sci. Educ..

[12] Schamberger G.P., Diez-Gonzalez F. (2004). Characterization of colicinogenic *Escherichia coli* strains inhibitory to enterohemorrhagic *Escherichia coli*. J. Food Prot..

[13] Cameron A., Zaheer R., Adator E.H., Barbieri R., Reuter T., McAllister T.A. (2019). Bacteriocin occurrence and activity in *Escherichia coli* isolated from bovines and wastewater. Toxins.

[14] Kozlovsky Y.E., Ovcharova A.N., Petrova V.A., Plugina I.V., Pustovalov S.A., Petnikov A.Y., Khomyakova T.I., Magomedova A.D., Chertovich N.F. (2012). Comparative effectiveness estimation of some probiotic strains of *Escherichia coli* in experimental disbiosis and toxicoinfection. Achievements of science and technology of the agro-industrial complex. NTP Anim. Husb. Feed. Prod..

[15] Hrala M., Bosák J., Micenková L., Křenová J., Lexa M., Pirková V., Tomáštíková Z., Koláčková I., Šmajs D. (2021). *Escherichia coli* strains producing selected bacteriocins inhibit porcine enterotoxigenic *Escherichia coli* (ETEC) under both in Vitro and in Vivo conditions. Appl. Environ. Microbiol..

[16] van Zyl W.F., Deane S.M., Dicks L.M.T. (2020). Molecular insights into probiotic mechanisms of action employed against intestinal pathogenic bacteria. Gut Microbes..

[17] Eiseul K., Seung-Min Y., Dayoung K., Hae-Yeong K. (2022). Complete genome sequencing and comparative genomics of three potential probiotic strains, *Lacticaseibacillus casei* FBL6, *Lacticaseibacillus chiayiensis* FBL7, and *Lacticaseibacillus zeae* FBL8. Front. Microbiol..

[18] Kuznetsova M.V., Mihailovskaya V.S., Remezovskaya N.B., Starčič Erjavec M. (2022). Bacteriocin-producing *Escherichia coli* isolated from the gastrointestinal tract of farm animals: Prevalence, molecular characterization and potential for application. Microorganisms.

[19] Cumsille A., Durán R.E., Rodríguez-Delherbe A., Saona-Urmeneta V., Cámara B., Seeger M., Araya M., Jara N., Buil-Aranda C. (2023). GenoVi, an open-source automated circular genome visualizer for bacteria and archaea. PLoS Comput. Biol..

[20] Jarocki P., Komoń-Janczara E., Młodzińska A., Sadurski J., Kołodzińska K., Łaczmański Ł., Panek J., Frąc M. (2023). Occurrence and genetic diversity of prophage sequences identified in the genomes of *L. casei* group bacteria. Sci. Rep..

[21] Wikivet 2012. Rat Haematology. https://en.wikivet.net/indexPhp?title-ReportHaematologysoldid=140051.

[22] Voitenko N.G., Makarova M.N., Zueva A.A. (2020). Variability of blood biochemical parameters and establishment of reference intervals in preclinical studies. Message 1: Rats. Lab. Anim. Sci. Res..

[23] Abrashova T.V., Gushchin Y.A., Kovaleva M.A., Rybakova A.V., Selezneva A.I., Sokolova A.P., Khodko S.V. (2013). Handbook. Physiological, Biochemical and Biometric Indicators of the Norm of Experimental Animals.

[24] Radwan M., Rashed RHamoda A.F., Amin A., Sakaya R.B. (2021). Experimental Infection with *E. coli* O157 in Rats and Its Toxic Effect, Biochemical and Histopathological Changes with Referee to Modern Therapy. Ann. Microbiol. Immunol..

[25] Upatissa S., Mitchell R.J. (2023). The “cins” of our fathers: Rejuvenated interest in colicins to combat drug resistance. J. Microbiol..

[26] Cursino L., Smajs D., Smarda J., Nardi R.M., Nicoli J.R., Chartone-Souza E., Nascimento A.M. (2006). Exoproducts of the *Escherichia coli* strain H22 inhibiting some enteric pathogens both in vitro and in vivo. J. Appl. Microbiol..

[27] Sassone-Corsi M., Nuccio S.P., Liu H., Hernandez D., Vu C.T., Takahashi A.A., Edwards R.A., Raffatellu M. (2016). Microcins mediate competition among Enterobacteriaceae in the inflamed gut. Nature.

[28] Unnerstad H.E., Lindberg A., Waller K.P., Ekman T., Artursson K., Nilsson-Öst M., Bengtsson B. (2009). Microbial aetiology of acute clinical mastitis and agent-specific risk factors. Vet. Microbiol..

[29] Saishu N., Ozaki H., Murase T. (2014). CTX-M-type extended-spectrum beta-lactamase-producing *Klebsiella pneumoniae* isolated from cases of bovine mastitis in Japan. J. Vet. Med. Sci..

[30] Kline K.A., Fälker S., Dahlberg S., Normark S., Henriques-Normark B. (2009). Bacterial adhesins in host-microbe interactions. Cell Host Microbe.

[31] Yoshida M., Thiriet-Rupert S., Mayer L., Beloin C., Ghigo J.-M. (2022). Selection for nonspecific adhesion is a driver of FimH evolution increasing *Escherichia coli* biofilm capacity. microLife.

[32] Bhoite S., van Gerven N., Chapman M.R., Remaut H. (2019). Curli Biogenesis: Bacterial Amyloid Assembly by the Type VIII Secretion Pathway. EcoSal Plus.

[33] Nesta B., Spraggon G., Alteri C., Moriel D.G., Rosini R., Veggi D., Smith S., Bertoldi I., Pastorello I., Ferlenghi I. (2012). FdeC, a novel broadly conserved *Escherichia coli* adhesin eliciting protection against urinary tract infections. mBio.

[34] Monteagudo-Mera A., Rastall R.A., Gibson G.R., Charalampopoulos D., Chatzifragkou A. (2019). Adhesion mechanisms mediated by probiotics and prebiotics and their potential impact on human health. Appl. Microbiol. Biotechnol..

[35] Papadimitriou K., Zoumpopoulou G., Foligné B., Alexandraki V., Kazou M., Pot B., Tsakalidou E. (2015). Discovering probiotic microorganisms: In vitro, in vivo, genetic and omics approaches. Front. Microbiol..

[36] Shahverdi S., Barzegari A.A., Bakhshayesh R.V., Nami Y. (2023). In-vitro and in-vivo antibacterial activity of potential probiotic *Lactobacillus paracasei* against *Staphylococcus aureus* and *Escherichia coli*. Heliyon.

[37] Melton-Celsa A.R. (2014). Shiga toxin (Stx) classification, structure, and function. Microbiol. Spectr..

[38] Veilleux S., Dubreuil J.D. (2006). Presence of *Escherichia coli* carrying the EAST1 toxin gene in farm animals. Vet. Res..

[39] Lorenz S.C., Son I., Maounounen-Laasri A., Lin A., Fischer M., Kase J.A. (2013). Prevalence of hemolysin genes and comparison of ehxA subtype patterns in Shiga toxin-producing *Escherichia coli* (STEC) and non-STEC strains from clinical, food, and animal sources. Appl. Environ. Microbiol..

[40] Wick R.R., Judd L.M., Holt K.E. (2019). Performance of neural network basecalling tools for Oxford Nanopore sequencing. Genome Biol..

[41] Kolmogorov M., Yuan J., Lin Y., Pevzner P.A. (2019). Assembly of long, error-prone reads using repeat graphs. Nat. Biotechnol..

[42] Wick R.R., Schultz M.B., Zobel J., Holt K.E. (2015). Bandage: Interactive visualisation of de novo genome assemblies. Bioinformatics.

[43] Tatusova T., DiCuccio M., Badretdin A., Chetvernin V., Nawrocki E.P., Zaslavsky L., Lomsadze A., Pruitt K.D., Borodovsky M., Ostell J. (2016). NCBI prokaryotic genome annotation pipeline. Nucleic Acids Res..

[44] Kleinheinz K.A., Joensen K.G., Larsen M.V. (2014). Applying the ResFinder and VirulenceFinder web-services for easy identification of acquired antibiotic resistance and *E. coli* virulence genes in bacteriophage and prophage nucleotide sequences. Bacteriophage.

[45] Wang M., Goh Y.X., Tai C., Wang H., Deng Z., Ou H.Y. (2022). VRprofile2: Detection of antibiotic resistance-associated mobilome in bacterial pathogens. Nucleic Acids Res..

[46] Seemann T. Abricate, Github. https://github.com/tseemann/abricate.

[47] Feldgarden M., Brover V., Haft D.H., Prasad A.B., Slotta D.J., Tolstoy I., Tyson G.H., Zhao S., Hsu C.H., McDermott P.F. (2019). Validating the AMRFinder tool and resistance gene database by using antimicrobial resistance genotype-phenotype correlations in a collection of isolates. Antimicrob. Agents Chemother..

[48] Blin K., Shaw S., Kloosterman A.M., Charlop-Powers Z., van Wezel G.P., Medema M.H., Weber T. (2021). antiSMASH 6.0: Improving cluster detection and comparison capabilities. Nucleic Acids Res..

[49] Blin K., Shaw S., Augustijn H.E., Reitz Z.L., Biermann F., Alanjary M., Fetter A., Terlouw B.R., Metcalf W.W., Helfrich E.J.N. (2023). antiSMASH 7.0: New and improved predictions for detection, regulation, chemical structures and visualisation. Nucleic Acids Res..

[50] Skinnider M.A., Johnston C.W., Gunabalasingam M., Merwin N.J., Kieliszek A.M., MacLellan R.J., Li H., Ranieri M.R.M., Webster A.L.H., Cao M.P.T. (2020). Comprehensive prediction of secondary metabolite structure and biological activity from microbial genome sequences. Nat. Commun..

[51] van Heel A.J., de Jong A., Song C., Viel J.H., Kok J., Kuipers O.P. (2018). BAGEL4: A user-friendly web server to thoroughly mine RiPPs and bacteriocins. Nucleic Acids Res..

[52] Wishart D.S., Han S., Saha S., Oler E., Peters H., Grant J.R., Stothard P., Gautam V. (2023). PHASTEST: Faster than PHASTER, better than PHAST. Nucleic Acids Res..

[53] Carattoli A., Zankari E., García-Fernández A., Larsen M.V., Lund O., Villa L., Møller F. (2014). In silico detection and typing of plasmids using PlasmidFinder and plasmid multilocus sequence typing. Antimicrob. Agents Chemother..

[54] Nikolaev Y.A. (2000). Regulation of adhesion in *Pseudomonas fluorescens* bacteria under the influence of distant intercellular interactions. Microbiology.

[55] Brilis V.I., Brilene T.A., Lentsner K.P., Lentsner A.A. (1986). Metodika izucheniya adgezivnogo processa mikroorganizmov. Lab. Delo..

[56] Eskova A.I., Andryukov B.G., Yakovlev A.A., Kim A.V., Ponomareva A.L., Obuhova V.S. (2022). Horizontal transfer of virulence factors by pathogenic Enterobacteria to marine saprotrophic bacteria during co-cultivation in biofilm. BioTech.

[57] Lenchenko E., Blumenkrants D., Sachivkina N., Shadrova N., Ibragimova A. (2020). Morphological and adhesive properties of *Klebsiella pneumoniae* biofilms. Vet. World.

[58] (1986). Guidelines for Accommodation and Care of Animals. Environment, Housing and Management; Directive 2010/63/EU of the European Parliament and of the Council on the Protection of Animals, and European Convention for the Protection of Vertebrate Animals Used for Experimental and other Scientific Purposes. ETS 123.

[59] Sarma P.R., Walker H.K., Hall W.D., Hurst J.W. (1990). Red Cell Indices. Clinical Methods: The History, Physical, and Laboratory Examinations.

