# Supplementary material for: Bacteriocin-Producing Escherichia coli Q5 and C41 with Potential Probiotic Properties: In Silico, In Vitro, and In Vivo Studies
Source: Int J Mol Sci. 2023 Aug 10;24(16):12636. doi: 10.3390/ijms241612636 (PMC10454217; doi:10.3390/ijms241612636)
Supplement: Supplementary file 1 [file ijms-24-12636-s001.zip › Figure S1.pdf]

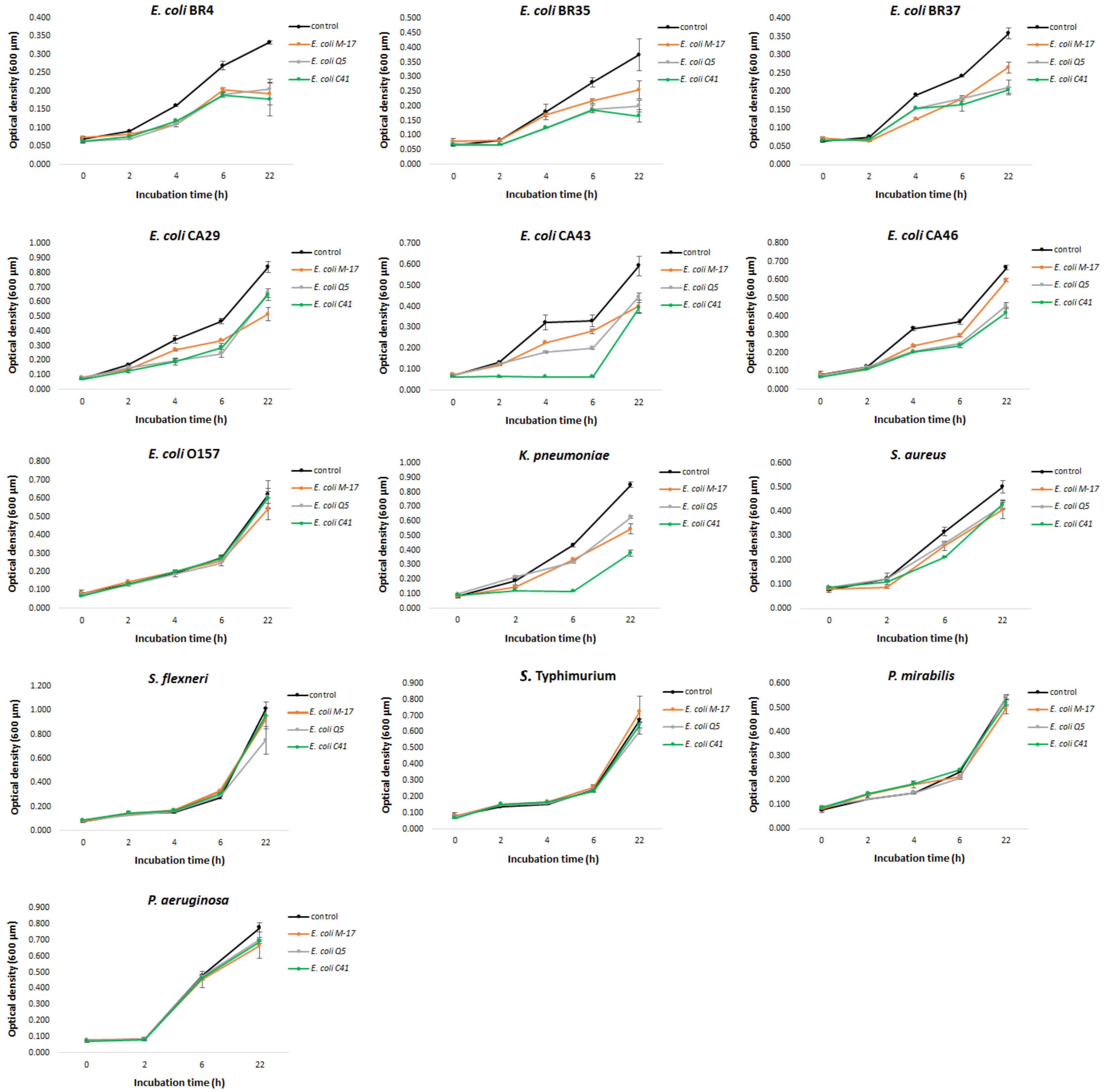

Figure S1. *In vitro* antimicrobial activity of cell-free supernatants of *E. coli* M-17, Q5 and C41 against indicated test strains in different times of incubation. The results are expressed as means ± mean standard deviations.
