# Supplementary material for: Bacteriocin-Producing Escherichia coli Q5 and C41 with Potential Probiotic Properties: In Silico, In Vitro, and In Vivo Studies
Source: Int J Mol Sci. 2023 Aug 10;24(16):12636. doi: 10.3390/ijms241612636 (PMC10454217; doi:10.3390/ijms241612636)
Supplement: Supplementary file 1 [file ijms-24-12636-s001.zip › Figure S2.pdf]

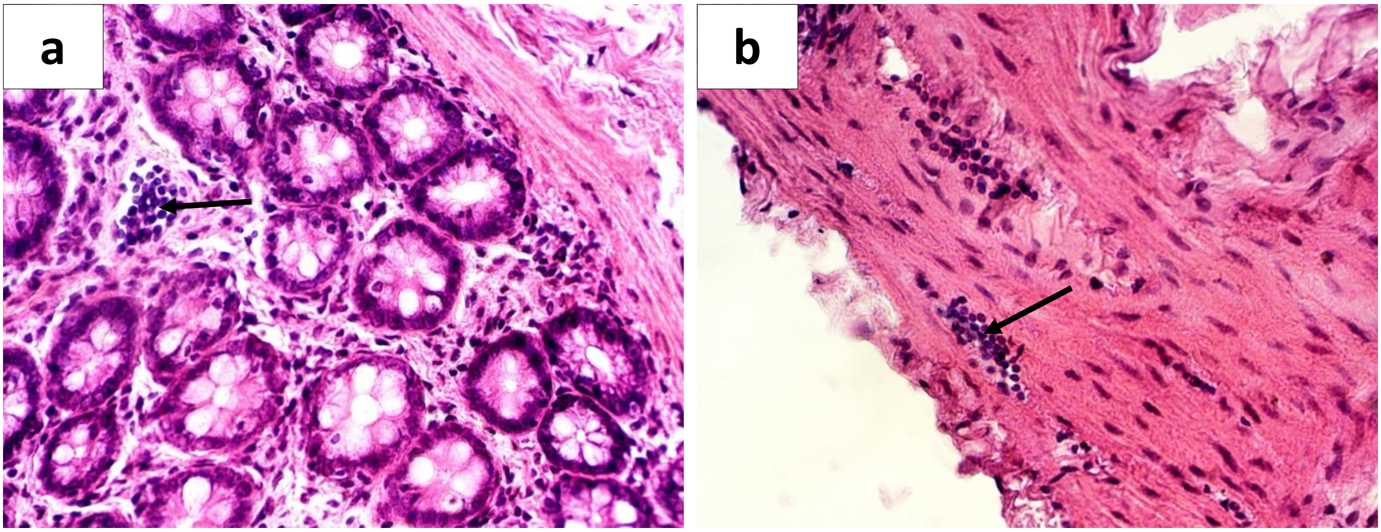

Figure S2. Hematoxylin-eosin stained colon sections of rats infected with *E. coli* C55 show accumulations of lymphocytes in the mucosa and submucosa layers (a, arrow, ×400), in the muscular layer (b, arrow, ×400).
