# Supplementary material for: Bacteriocin-Producing Escherichia coli Q5 and C41 with Potential Probiotic Properties: In Silico, In Vitro, and In Vivo Studies
Source: Int J Mol Sci. 2023 Aug 10;24(16):12636. doi: 10.3390/ijms241612636 (PMC10454217; doi:10.3390/ijms241612636)
Supplement: Supplementary file 1 [file ijms-24-12636-s001.zip › Table S1, S2, S3.pdf]

Supplementary Table S1. General sequencing and genomic statistics for *E. coli* Q5 and C41.

	***E. coli* Q5**	***E. coli* C41**
BioProject accession no.	PRJNA980458	
BioSample accession no.	SAMN35637330	SAMN35637331
SRA accession no. (ONT reads)	SRR24834173	SRR24834172
GenBank accession no.	CP127255-CP127260	CP127252-CP127254
Total reads length (Mb)	867.7	684.3
No. of reads	88676	62481
Reads N50 (kb)	16.4	19.1
Total genome length, bp	5,267,038	5,165,039
Chromosome length, bp	4,948,409	5,037,330
GC content %	51.0	51.3
Number of CDS	4945	4878
Number of rRNA	22	22
Number of tRNA	89	89
Number of tmRNA	1	1
Number of episomes	5	2

Supplementary Table S2. Description of prophages detected in the genomes of the *E. coli* Q5 and C41 strains using PHASTEST.

Strain	Replicon	Coordinates, bp	Phage & hypothetical proteins, %	Completeness	Similar to
Q5	Chromosome	1198161:1214642	95,20%	intact	PHAGE_Burkho_phiE255_NC_009237
Q5	Chromosome	1227480:1282768	95,30%	intact	PHAGE_Entero_mEp460_NC_019716
Q5	Chromosome	2586661:2622614	97,90%	intact	PHAGE_Klebsi_4LV2017_NC_047818
Q5	Chromosome	2906727:2969625	92,80%	intact	PHAGE_Entero_lambda_NC_001416
Q5	Chromosome	3717261:3769714	98,20%	intact	PHAGE_Entero_SfV_NC_003444
Q5	Chromosome	4210500:4252420	96,40%	intact	PHAGE_Entero_P88_NC_026014
Q5	pQ502	522:97406	100%	intact	PHAGE_Salmon_SJ46_NC_031129
C41	Chromosome	1072440:1086513	93,70%	intact	PHAGE_Entero_P4_NC_001609
C41	Chromosome	1176160:1215640	94,50%	intact	PHAGE_Entero_SfV_NC_003444
C41	Chromosome	1581859:1593071	91,60%	intact	PHAGE_Entero_P4_NC_001609
C41	Chromosome	1871516:1917394	96,40%	intact	PHAGE_Entero_lambda_NC_001416
C41	Chromosome	2142922:2185862	98,40%	intact	PHAGE_Entero_Sf101_NC_027398
C41	Chromosome	2328774:2365405	97,80%	intact	PHAGE_Entero_fiAA91_ss_NC_022750
C41	Chromosome	2805201:2853662	91%	intact	PHAGE_Entero_lambda_NC_001416
C41	Chromosome	3020367:3026520	85,70%	intact	PHAGE_Escher_EC6098_NC_048874
C41	Chromosome	3054115:3122513	72,90%	intact	PHAGE_Entero_lambda_NC_001416
C41	Chromosome	3512532:3541014	97,50%	intact	PHAGE_Escher_vB_EcoM_ECOO78_NC_041926
C41	Chromosome	3541320:3570751	100%	incomplete	PHAGE_Pectob_ZF40_NC_019522

Supplementary Table S3. Description of episomes found in the *E. coli* Q5 and C41.

Episome	Length, bp	Copy-number (Flye)	Coverage/ Identity, %	Best hit (BLASTn vs nt)	Incompatibility group (PlasmidFinder-2.0)	Plasmid transfer system
pQ501	137,557	2	81/99.9	pH22_122286 (CP120564.1)	IncFIB, IncFII	F-type (tra) conjugation system
pQ502	98,317	1	89/99.6	Punavirus P1 (MH422554.1)	p0111	-
pQ503	58,014	2	100/99.7	pNMBU-W12E19_04 (CP042888.1)	IncI2	P-type (virB) conjugation system
pQ504	17,107	1	100/99.9	pNGF1_pCol_let_like (CP016009.1)	ColE1	mobilization system (MobC)
pQ505	7,791	89	100/99.9	unnamed plasmid 1 CP077681.1	?	mobilization system (MobA/MobL)
pC4101	114,534	1	83/99.9	p1 (CP083264.1)	IncFIB, IncFIC	F-type (tra) conjugation system
pC4102	13,295	1	100/99.9	pFF2-1h-4 (CP097202.1)	ColE1	mobilization system (MobC-like)
